# Routine ICU Surveillance after Brain Tumor Surgery: Patient Selection Using Machine Learning

**DOI:** 10.3390/jcm13195747

**Published:** 2024-09-26

**Authors:** Jan-Oliver Neumann, Stephanie Schmidt, Amin Nohman, Paul Naser, Martin Jakobs, Andreas Unterberg

**Affiliations:** Department of Neurosurgery, University Hospital Heidelberg, 69120 Heidelberg, Germany

**Keywords:** craniotomy, complications, postoperative surveillance, ICU, machine learning

## Abstract

**Background/Objectives:** Routine postoperative ICU admission following brain tumor surgery may not benefit selected patients. The objective of this study was to develop a risk prediction instrument for early (within 24 h) postoperative adverse events using machine learning techniques. **Methods:** Retrospective cohort of 1000 consecutive adult patients undergoing elective brain tumor resection. Nine events/interventions (CPR, reintubation, return to OR, mechanical ventilation, vasopressors, impaired consciousness, intracranial hypertension, swallowing disorders, and death) were chosen as target variables. Potential prognostic features (*n* = 27) from five categories were chosen and a gradient boosting algorithm (XGBoost) was trained and cross-validated in a 5 × 5 fashion. Prognostic performance, potential clinical impact, and relative feature importance were analyzed. **Results:** Adverse events requiring ICU intervention occurred in 9.2% of cases. Other events not requiring ICU treatment were more frequent (35% of cases). The boosted decision trees yielded a cross-validated ROC-AUC of 0.81 ± 0.02 (mean ± CI95) when using pre- and post-op data. Using only pre-op data (scheduling decisions), ROC-AUC was 0.76 ± 0.02. PR-AUC was 0.38 ± 0.04 and 0.27 ± 0.03 for pre- and post-op data, respectively, compared to a baseline value (random classifier) of 0.092. Targeting a NPV of at least 95% would require ICU admission in just 15% (pre- and post-op data) or 30% (only pre-op data) of cases when using the prediction algorithm. **Conclusions:** Adoption of a risk prediction instrument based on boosted trees can support decision-makers to optimize ICU resource utilization while maintaining adequate patient safety. This may lead to a relevant reduction in ICU admissions for surveillance purposes.

## 1. Introduction

Historically, routine admission to an intensive care unit (ICU) following brain tumor surgery has been carried out in many institutions, although the indication and effectiveness remained unclear [1,2,3,4,5,6,7,8]. Continuous advances in neurosurgery and anesthesiology have resulted in reduced invasiveness, reduction of time spent under general anesthesia, and consequently, faster postoperative neurologic recovery. It has been demonstrated in various studies that the complication rate of brain tumor resection is low nowadays, even for the elderly [9,10]. Consequently, the necessity of ICU admission as a routine measure has been questioned by many authors in recent years.

Systematic reviews of the available literature concluded that routine postoperative admission of post-craniotomy patients to the ICU “*may not benefit carefully selected patients*” [8] and that “*non-ICU care pathways […] represent a meaningful opportunity to improve care value*” [11]. Both authors advised caution when considering and implementing non-ICU pathways and stressed the importance of proper patient selection.

Additionally, the COVID-19 pandemic has demonstrated the competition of various medical specialties for limited ICU resources and has underlined the need for an objective and fair risk assessment for ICU admission following brain tumor surgery. Motivated by these opportunities to improve patient care and resource utilization, risk prediction scores have been derived and non-ICU postoperative pathways have been evaluated in smaller cohorts [12,13,14,15,16,17,18,19].

When considering the implementation of an alternative pathway, it must be kept in mind that equipment and staffing of various non-ICU treatment units like intermediate-care (IMC) or postoperative-care (PACU) units vary considerably between healthcare systems and individual institutions. Consequently, any non-ICU postoperative protocol must be tailored to the individual situation, and an objective risk assessment remains the essential factor for the efficacy and safety of non-ICU pathways.

Finding a tailored solution for the problem at hand, we recently conducted and published an external validation and critical appraisal of the two most promising risk prediction scores [20]. Our analysis demonstrated rather disappointing performance and limited practical clinical use of these scores in our setting. Major limiting factors were narrow scope (only supratentorial tumors), specific postoperative ICU-like surveillance protocols, and the mandatory use of intraoperative data. Scores relying on data only available after surgery is concluded cannot be used for preoperative scheduling decisions and consequently are of limited use in a clinical setting.

From our assessment of the available literature, we had to conclude that the methods used so far to deduce a risk prediction instrument (uni- or multivariate logistic regression from a small set of independent variables) are insufficient to attain satisfactory results in this complex and non-linear problem. It has been shown that machine learning techniques might be more suitable to recognize patterns of features in craniotomy patients at risk of early complications [21].

As a consequence, we decided to develop a risk prediction instrument that meets the following requirements: it should be applicable to most brain tumor resection cases (supra- and infratentorial), it should be narrow in scope to predict only early (within 24 h) postoperative adverse events requiring ICU treatment, and it should be suitable for pre- and postoperative ICU-admission decisions.

In order to pursue this objective, we trained a state-of-the-art implementation of boosted decision trees on a cohort of brain tumor cases undergoing elective resection at our institution.

## 2. Materials and Methods

The patient cohort consists of 1000 consecutive cases of elective brain tumor resection in adults performed at our institution between January 2019 and July 2020. Cases with craniotomy for cerebral aneurysms, microvascular decompression, and pituitary adenomas were excluded.

The decision for admission to the ICU was made prior to surgery by the joint judgment of the neurosurgeon and the anesthesiologist. In the cohort, 917 (92%) patients were admitted to the ICU, and 83 (8%) individuals were scheduled for direct transfer to the neurosurgical floor after short surveillance in the PACU.

After a careful review of the available literature, we selected 27 candidate features from five categories (*demographics, preoperative conditions, past medical history, tumor characteristics on MRI, and intraoperative data*) that were of potential prognostic value for the prediction of adverse events. The majority (23) of these features were available before the start of the procedure.

After consideration of the literature and the work done by other authors, we selected and defined nine events and/or interventions by expert opinion (*CPR, reintubation, return to OR, mechanical ventilation, vasopressors, impaired consciousness, intracranial hypertension, swallowing disorders, and death, Table 1*) that require treatment on an ICU. The goal of the prediction instrument was to assess the likelihood of any of these events within 24 h after surgery using only the prognostic features given above.

Other noteworthy events that might require specific actions or an elevated level of staff attention (any cranial nerve deficit, hemiparesis ≤ 3/5, administration of mannitol, postoperative CT scan, any seizure or intravenous blood pressure medication) were recorded but not considered to require mandatory ICU treatment/surveillance.

All data were retrospectively extracted from clinical records. Preoperative MR images were reviewed. Furthermore, the anesthesia and subsequent ICU and/or neurosurgical floor reports were searched for any intra- or postoperative adverse events following our definitions given in Table 1. A routine postoperative CT scan was not performed at our institution. There was no missing data with respect to our study.

**Table 1:** Definition of postoperative ICU events.

ICU Event	Definition
CPR	Any CPR delivered
Reintubation	Reintubation for any reason other than revision surgery
Return to OR	Any surgery due to complications within 24 h
Mechanical ventilation	Any postoperative ventilation > 4 h
Vasopressors	Continuous application of more than 0.4 mg/h norepinephrine
Impaired consciousness	GCS < 13
Intracranial hypertension	CSF drainage or administration of mannitol due to ICP > 20 mmHg
Swallowing Disorder	Impaired swallowing requiring a gastral tube or parenteral nutrition
Death in the perioperative period	Any death within 48 h post-surgery

For the classification and risk stratification task, a supervised gradient boosting technique was used. Gradient boosting uses an ensemble of decision trees that are iteratively optimized to minimize the loss function. The XGBoost [22] framework (version 1.7.6, https://xgboost.ai, accessed on 13 January 2024) was used (cf. Appendix A for technical details).

According to clinical experience and the literature [8,9,12,13], a skewed distribution towards cases without postoperative adverse events was expected. As the exact rate would depend on the breadth of the definition of adverse events, an initial retrospective pilot survey of 100 cases with our definition was performed that yielded an estimated rate of 10% postoperative adverse events. Given the variety of features and their possible combinations, we concluded that each split during training and validation should contain 10 to 20 positive cases to ensure that proper boosting and validation of the trees is feasible. Therefore, we chose a large sample size of 1000 patients resulting in approximately 20 positive cases within each split (5-fold cross-validation).

The prognostic performance of the score was assessed by calculating the area under the curve of the receiver operating characteristic (AUC-ROC) and the area under the curve of the precision-recall curve (AUC-PR). Calibration of the final models was estimated with reliability plots and calculation of the Brier score.

All confidence intervals were calculated using a t-distribution with an α<5% (CI_95_). Statistical analyses were conducted with the SciPy [23] package (version 1.11.2).

## 3. Results

### 3.1. Summary of Prognostic Features

The study included 552 female (55%) and 448 male (45%) patients aged 18 to 88 (mean 57 ± 15 SD) years. The overwhelming majority (94%) were assigned to ASA-PS (American Society of Anesthesiologists Performance Score) classes II and III. Arterial hypertension (41%) and diabetes mellitus (11%) were the two most common comorbidities. Twenty-six percent of patients had a history of prior craniotomy while 23% had a history of epileptic seizures.

Craniotomy for removal of meningiomas (36%), cerebral metastases (19%), or glioblastoma (16%) made up almost three-quarters of all procedures. The tumor location was supratentorial in 78% of cases and infratentorial in 22%. Intraventricular tumors accounted for 25 (3%) cases.

Table 2 gives a summary of the prognostic features in the cohort.

### 3.2. Postoperative Events

During postoperative surveillance on the ICU (92%, *n* = 917) or PACU/neurosurgical floor (8%, *n* = 83), adverse events requiring ICU intervention (or readmission) occurred in 92 cases (9.2%) (Table 3). The total number of events was 149.

Two postoperative adverse events occurred after the patient had already been transferred from the PACU to the neurosurgical floor: one case of arterial hypotension requiring vasopressor therapy and one case of neurological deterioration with hemiparesis and reduced vigilance. A subsequent CT scan revealed an intracerebral hematoma requiring operative revision. Other noteworthy events or conditions not requiring ICU intervention were more frequent and occurred in about one-third of the cases (351 cases, 540 total events). A postoperative CT scan was the most common event (99) in this group.

### 3.3. Classification Performance

**Figure 1:**
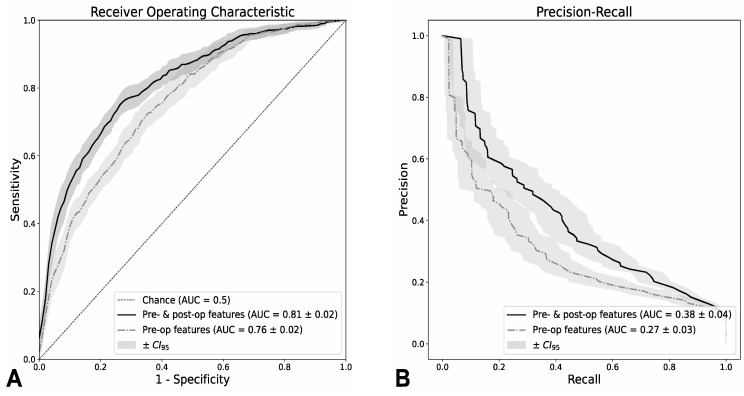
Prognostic performance. Pre- and post-op features yield a ROC-AUC 0.81 ± 0.02 (mean ± CI_95_) in 5-times repeated 5-fold cross-validation (*p* < 0.01). Using only pre-op features, ROC-AUC still scores at 0.76 ± 0.02 (*p* < 0.001, (**A**)). As the underlying class distribution is clearly skewed towards the negative class (1:9), the AUC of the precision-recall curve was expected to be lower than AUC-ROC. In the case of pre- and post-op data, AUC-PR was 0.38 ± 0.04, and 0.27 ± 0.03 for pre-op data only (both *p* < 0.001, (**B**)). Compared to the baseline value of a random classifier (0.09), these numbers represent a 3-fold increase in the baseline value and represent a good classification performance.

The trained ensemble of boosted decision trees yielded an area under the curve (AUC) of the receiver operating characteristic (ROC) of 0.81 ± 0.02 (mean ± CI_95_) when using pre- and post-op data. In the case of using only pre-op data (operating room scheduling decisions), ROC-AUC was lower at 0.76 ± 0.02 (Figure 1A) as expected.

As the underlying class distribution was clearly skewed towards the negative class (1:9), the area under the curve of the precision-recall curve (AUC-PR) was unsurprisingly lower than AUC-ROC which was distribution-agnostic. In the case of pre- and post-op data, AUC-PR was 0.38 ± 0.04, while for pre-op data only, it was 0.27 ± 0.03 (Figure 1B). Compared to the baseline value (random decision) of 0.09, these numbers show a 3-fold increase to the baseline and represent a good classification performance.

When considering only supratentorial cases (*n* = 777), the models reached an AUC-ROC of 0.75 ± 0.03 (pre- and post-op) and 0.74 ± 0.02 (pre-op only). AUC-PR was scored at 0.31 ± 0.04 for pre- and post-op, and 0.29 ± 0.04 for pre-op only with a baseline value of 0.11 (Figure S1).

### 3.4. Calibration and Admission Rate/Safety Tradeoff

Boosted trees usually do not produce well-calibrated posterior probabilities. They tend to predict probabilities very conservatively, i.e., they are biased towards the center and avoid the extremes. The reliability plot of the raw model output (Supplemental Figure S2, black curve) shows a distribution of predicted probabilities that is displaced towards the right (systematic overestimation, Brier score 0.09 ± 0.01 in 5 × 5 cross-validation). This phenomenon is well-known when training with an unbalanced dataset with very few positives. Fitting an isotonic regressor that maps the output of the classifier to a calibrated probability in [0, 1] has been recommended to improve calibration of decision tree models [24]. Supplemental Figure S2 (gray, interrupted curve) shows a clear improvement of predicted probabilities after calibration in this manner (Brier score 0.07 ± 0.01).

**Figure 2:**
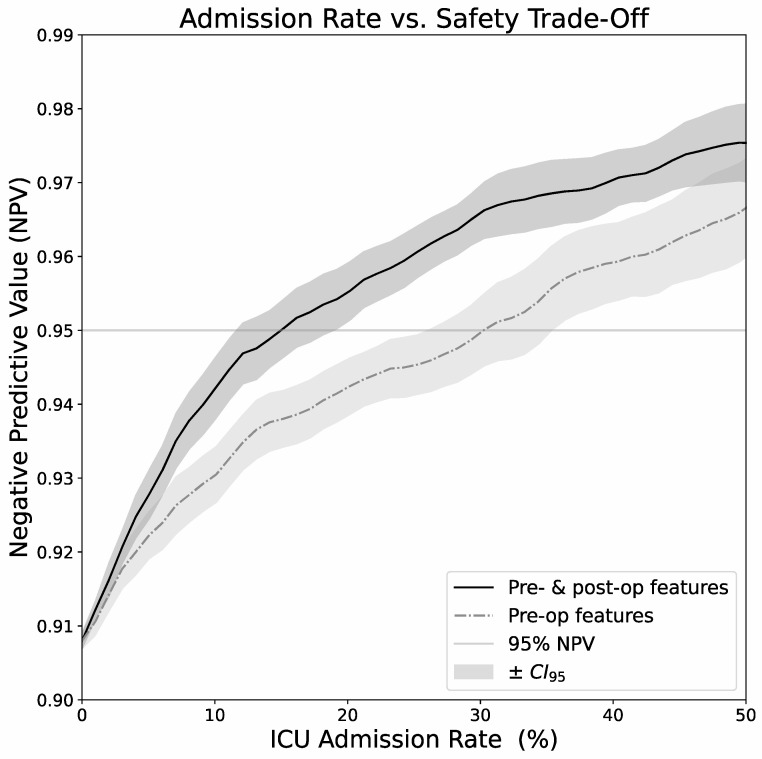
Relationship between negative predictive value (NPV) and ICU admission rate as a function of the selected threshold. With pre- and post-op data, targeting an NPV of at least 95% requires ICU admission in 15% of cases. Using only preoperative data, approximately 30% of cases were selected for surveillance at the ICU. Higher target NPVs lead to continuously rising ICU admission rates.

Choosing an ICU-event probability threshold for an actual planned ICU admission requires a tradeoff between admission rate (ICU resource utilization) and patient safety. Figure 2 demonstrates the relationship between negative predictive value (NPV) and ICU admission rate as a function of the selected threshold. With pre- and post-op data, targeting a NPV of at least 95% requires ICU admission in 15% of cases. Using only preoperative data, approximately 30% of cases were selected for surveillance at the ICU. Higher target NPVs lead to continuously rising ICU admission rates. As a matter of fact, almost perfect safety (i.e., approaching 100% NPV) is only attainable by prohibitive ICU admission rates of more than 90%.

### 3.5. Relative Importance of Prognostic Features

**Figure 3:**
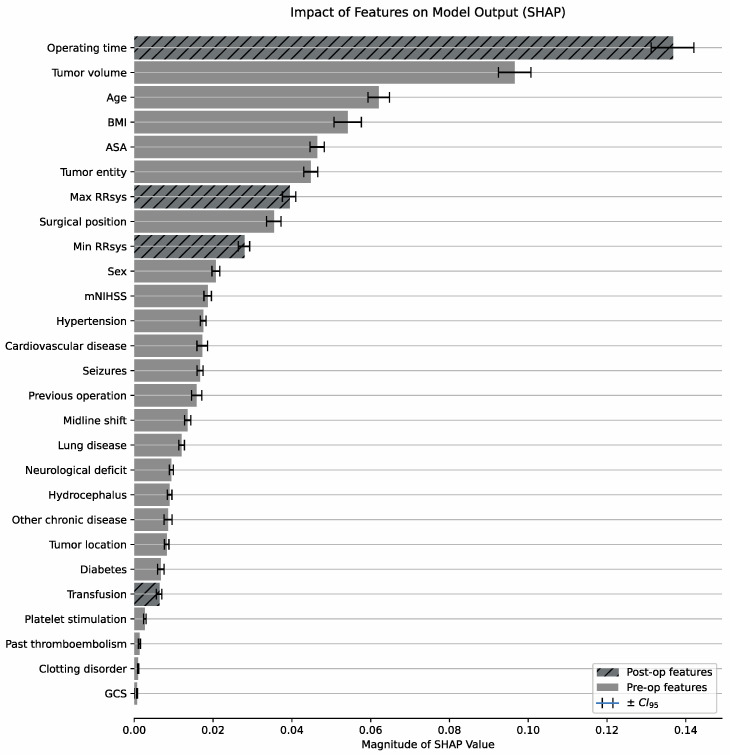
Relative contribution of features to the model output.

SHAP (SHapley Additive exPlanations) is a method to explain individual predictions of tree-based models and are based on Shapley values used in game theory. SHAP values have been shown to be a way to consistently attribute feature importance in tree-based machine learning models [25]. The XGBoost implementation used in this study is capable of directly providing SHAP values for the trained models. The magnitude of the mean SHAP values for a given feature is indicative that a given feature is more important for generating a prediction than features with lower values (feature importance, Figure 3). The features found with high SHAP values correspond well with known risk factors for postoperative complications (top five: operating time, tumor volume, age, BMI, and ASA class) from clinical experience and previous studies. A more detailed analysis of the interactions between individual features has been performed and is shown in Figures S3 and S4.

## 4. Discussion

In our retrospective investigation, we used a fast and robust machine learning method (gradient boosted trees) to identify patients at elevated risk of postoperative events requiring ICU intervention following elective brain tumor surgery.

We were able to score a ROC-AUC of 0.81 ± 0.02 (pre- and post-op data) and 0.76 ± 0.02 (pre-op data only). In the case of pre- and post-op data, AUC-PR was 0.38 ± 0.04, and 0.27 ± 0.03 for pre-op data only (Figure 1B). Compared to the random baseline value of 0.092, these numbers show a 3-fold increase to the baseline and represent a good classification performance.

Considering that perfect prediction of future events is not within reach, a tradeoff between negative predictive value and ICU admission rate is necessary. The choice of the minimum acceptable negative predictive value is of ethical, socio-economic, and medico-legal nature and might vary between individual institutions and healthcare systems.

Since a false-negative decision (i.e., not admitting a patient to ICU) puts a patient at risk but not necessarily causes irreversible harm, we would choose an NPV of 95%. The minimum goal of 95% NPV would lead to a planned ICU-admission of approximately 30% of patients when using only preoperative data. At the end of surgery, when more data is available, a more informed decision could be made that would consider only 15% of patients in need of ICU admission for further surveillance. The goal of our endeavor is to help decision-makers to optimize ICU resource utilization, increase fairness among individual patients and medical (sub-)specialties while maintaining adequate patient safety.

The clinical relevance of finding an objective risk prediction instrument for postoperative ICU admission following brain tumor surgery is supported by the fact that several authors have already proposed solutions for this problem [12,13,14,15,16].

Franko et al. [16] derived a simple score with logistic regression of only three features, but scoring is limited to supratentorial tumors and considers only early complications within 4 h of surgery as independent variable, effectively relocating postoperative surveillance from the ICU to the PACU. Furthermore, training and validation cohorts were too small (300 patients in total) when taking the low incidence of postoperative complications into account. Another scoring system very similar in scope and sample size (*n* = 400) was put forward by Munari et al. [13] in 2022, again relying on prolonged postoperative surveillance for 6–8 h in the PACU.

Rozeboom et al. [14] published a multivariable prediction model for postoperative intensive care unit stay in a broad surgical population. It included 2616 neurosurgical cases of all kinds including spinal cases. The sample included outpatient and emergency surgery cases. The authors admitted that ICU referral in the neurosurgical subgroup (72% no ICU use, 28% ICU use) was based mostly on surgeon’s request rather than an objective assessment of the need for it. Schipmann et al. [15] took a heterogenous (including biopsies, pituitary, and spinal tumors) cohort of 811 cases and derived the “SOS”-score by multivariate logistic regression and stratified patients in low, medium, and high risk groups based on number of secondary diagnoses, BMI, and operating time.

The CranioScore derived by Cinotti et al. [12] represents the most substantial and rigorous attempt to derive a risk score for ICU-worthy events using logistic regression from a retrospective single-center database of 1094 patients. Validation in a prospective, multi-center cohort (*n* = 830) found a ROC-AUC of 0.70 ± 0.06 and a rather disappointing ROC-AUC of 0.65 when tested in a retrospective 1000 patient cohort from our hospital for evaluation purposes [20].

Other groups followed a ‘protocol-driven’ approach for the problem at hand and defined clinical, non-ICU pathways for managing selected patients classified as ‘low-risk’. The definition of ‘low-risk’ and the measures taken for non-ICU-admitted patients were based on expert opinion and vary considerably between published protocols.

The “Non-Intensive CarE (NICE)” protocol is based on nine preoperative and six postoperative ICU/non-ICU criteria and limited to extra-axial tumors or patients and procedures ‘deemed (…) to be low-risk and suitable for non-ICU postoperative care’ by the attending neurosurgeon [26]. In a 3-year assessment after adoption of the protocol, 534 out of 2535 (21%) craniotomies were eligible for the non-ICU pathway and ICU admission rate subsequently dropped from 57% to 42% compared to a historic control of 2302 patients [6].

The “No ICU—Unless (NIU)” protocol reversed the tradition of routine ICU admission to tentative management of supratentorial, elective tumor surgeries on the neurosurgical floor unless neurosurgeon or anesthesiologist decided otherwise [27]. After adoption of the protocol, a decline in ICU admission rate from 64% to 24% was noted in a cohort study of 109 patients with equivalent complication rates as in the historical controls (*n* = 107). Qasem et al. [28] reported similar results of the NIU protocol in a matched-pair analysis of 171 patients.

Hoffman et al. [19] recently demonstrated a reduced length of stay in a control study of a subset of patients undergoing supratentorial craniotomy that were directly transferred from the PACU to the floor.

Both approaches—whether protocol-driven or data-driven—ultimately still require an individual decision by a responsible physician. To minimize the risk of irreversible harm, this decision will also depend on the neurosurgical expertise and staffing of any ‘alternative’ postoperative care unit, whether this would be the PACU, a step-down (IMC) unit or the neurosurgical floor. In our opinion, the quality and staffing of these units are very important and should receive proper consideration in any scientific and clinical discussions.

At the current stage, our first approach to risk stratification and patient selection using machine learning still carries important limitations that must be addressed.

The prediction model is derived from a retrospective cohort and has not been validated in independent cohorts and different clinical settings. Nevertheless, cross-validation (5-fold, 5-times repeated) has shown small confidence intervals in the performance parameters of the hold-out data, suggesting good performance in yet unseen data

Additionally, our selection and definition of ICU- and non-ICU worthy events, although very similar to the decisions in the studies discussed above, remains subjective and is based on the clinical experience and reasoning of the authors of this study. We have tried to give a reasonable distinction between ICU-worthy and other noteworthy events (those events that we consider to be salvageable in a non-ICU setting), but the labeling of the cases should be further operationalized in future studies.

Taking the great variance of quality, staffing, and individual experiences into account, a broad discussion between clinicians can be expected here and it seems unlikely that a consensus can be found easily. As an example, the intravenous administration of antihypertensive drugs might require invasive blood pressure monitoring in some institutions while other clinicians would feel that this can be done safely with frequent but non-invasive blood pressure monitoring.

Finally, it has to be kept in mind that there might be an underreporting of adverse events for those patients that went to the neurosurgical floor. This is true not only for our study, but for the whole class of studies mentioned above. In our sample, reflecting our more traditional approach, 92% of the patients were monitored on the ICU, not on a neurosurgical floor. Comparing the actual event rates between patients going to the ICU and the floor, we found no significant differences.

This work is based on a large cohort of 1000 cases, only second in size to the 1094 cases in the training cohort of Cinotti et al. [12], but uses boosted trees rather than logistic regression. Boosted trees represent a well-established and robust machine learning technique that is designed to work well with categorical and even missing data found in a clinical setting. Niftrik et al. [21] have already shown that boosted trees are superior to conventional statistical methods in their analysis of 688 craniotomy patients.

It has to be stressed that the preliminary work presented here is not ready for clinical use yet. Starting from that point, once more data and models come available, confidence in AI applications in these complex clinical settings will improve and could also provide further insights (e.g., feature importance) to adapt future clinical guidelines. If machine learning methods such as the one presented here are properly embedded in a clinical protocol for the managing of non-ICU cases, the utilization of the ICU for postoperative surveillance of tumor resection patients could be reduced while maintaining adequate patient safety. In the future, these models can be modified and extended to further differentiate between different levels of postoperative surveillance levels such as ICU, IMC, and neurosurgical floor.

## 5. Conclusions

We suggest that the development of a risk prediction instrument based on boosted decision trees can support decision-makers to optimize ICU resource utilization, and increase fairness among individual patients and medical (sub-)specialties while maintaining adequate patient safety. If properly validated in prospective studies and embedded in a clinical protocol, this may lead to a relevant reduction in ICU admissions for surveillance purposes and could also provide insights for adapted clinical guidelines.

## Figures and Tables

**Table 2:** Prognostic features.

Prognostic Features (27 Total)	Values
Demographics (3)	
Age	18–88 years (57 ± 15) ^1^
Sex	55% female, 45% male
BMI	13–46 (26 ± 5) *
Preoperative conditions (4)	
ASA	I (6%) II (58%) III (36%) IV (1%) V (0%)
mNIHSS	0–11, median 0, IQR 0–1
GCS	11–15, median 15, IQR 15–15
Any neurologic deficit	63%
Past medical history (10)	
Diabetes mellitus	11%
Arterial Hypertension	41%
Clotting disorder	2%
Thromboembolism	4%
Seizures	26%
Prior neurosurgery	23%
Antiplatelet or anticoagulation meds	14%
Cardiovascular disease	14%
Lung disease ^1^	56%
Other chronic diseases	14%
Tumor characteristics (5)	
Hydrocephalus on MRI	6%
Location of tumor	78% supratentorial 22% infratentorial
Midline shift (>3 mm) on MRI	19%
Suspected entity	36% meningioma, 19% metastasis, 16% glioblastoma, 28% other
Tumor volume ^2^	1–272 mL (22 ± 31)
Intra- and postoperative data (4 + 1)	
Positioning	supine 67% prone 13%, lateral 7% sitting 13%
Duration of surgery (min)	20–740 min (236 ± 102)
Maximum systolic blood pressure (mmHg)	90–180 (135 ± 16)
Minimum systolic blood pressure (mmHg)	50–140 (100 ± 10)
Transfusion of red blood cells, platelets or plasma	2%

* Mean + SD; Abbreviations: BMI: Body Mass Index; ASA: American Society of Anesthesiologists Performance Score; mNIHSS: modified National Institute of Health Stroke Scale; GCS: Glasgow Coma Scale; MRI: Magnetic Resonance Imaging. ^1^ relevant (as noted by the premedication anesthesiologist) restriction or obstruction, lung tumors, or pneumonia. ^2^ as given in the radiology report (measurement in the preoperative MRI).

**Table 3:** Postoperative events (within 24 h).

Postoperative Events	No. of Cases (*n* = 1000)
ICU events	149
Cases with events	92 (9.2%)
CPR	4 (0.4%)
Return to OR	12 (1.2%)
Continued mechanical ventilation	25 (2.5%)
Vasopressors	22 (2.2%)
Impaired consciousness	34 (3.4%)
Intracranial hypertension	22 (2.2%)
Swallowing Disorder	17 (1.7%)
Death in the perioperative period	0 (0.0%)
Other events	540
Cases with events	351 (35.1%)
Any cranial nerve deficit	72 (7.2%)
Hemiparesis (≤3/5)	46 (4.6%)
Administration of mannitol	47 (4.7%)
Postoperative CT scan	99 (9.9%)
Seizure	42 (4.2%)
I.V. blood pressure medication	47 (4.7%)

## Data Availability

The source data contains pseudonymized patient data protected from public disclosure. It can be made available in anonymized form upon reasonable request and pending clearance by the institutional review board of the University of Heidelberg. Computer code can be made available upon reasonable request.

## Supplementary Materials

The following supporting information can be downloaded at https://www.mdpi.com/article/10.3390/jcm13195747/s1, Figure S1: Prognostic performance for supratentorial cases; Figure S2: Reliability and calibration; Figure S3: Summary plot of SHAP values; Figure S4: SHAP values for selected features.

## Appendix A

The boosted trees were trained, optimized, and validated using 5-times repeated (randomly reshuffled), 5-fold cross-validation for a total of 25 runs.

In each cycle, the data was split in an 80% training/optimization and 20% hold-out validation set. The trees were trained and hyperparameters optimized using another 5-fold cross-validation on each training set (800 cases) using the Optuna [29] framework (version 3.3.0, https://optuna.org, accessed on 13 January 2024, tree-structured Parzen estimator, 5000 iterations per run). Diagnostic performance was validated in the hold-out set (200 cases) and confidence intervals for the metrics were calculated for the 25 runs. Additional machine learning tasks were performed with the scikit-learn [30] package (version 1.2.2).
