# Supplementary material for: Routine ICU Surveillance after Brain Tumor Surgery: Patient Selection Using Machine Learning
Source: J Clin Med. 2024 Sep 26;13(19):5747. doi: 10.3390/jcm13195747 (PMC11477277; doi:10.3390/jcm13195747)
Supplement: Supplementary file 1 [file jcm-13-05747-s001.zip › Figure S1.pdf]

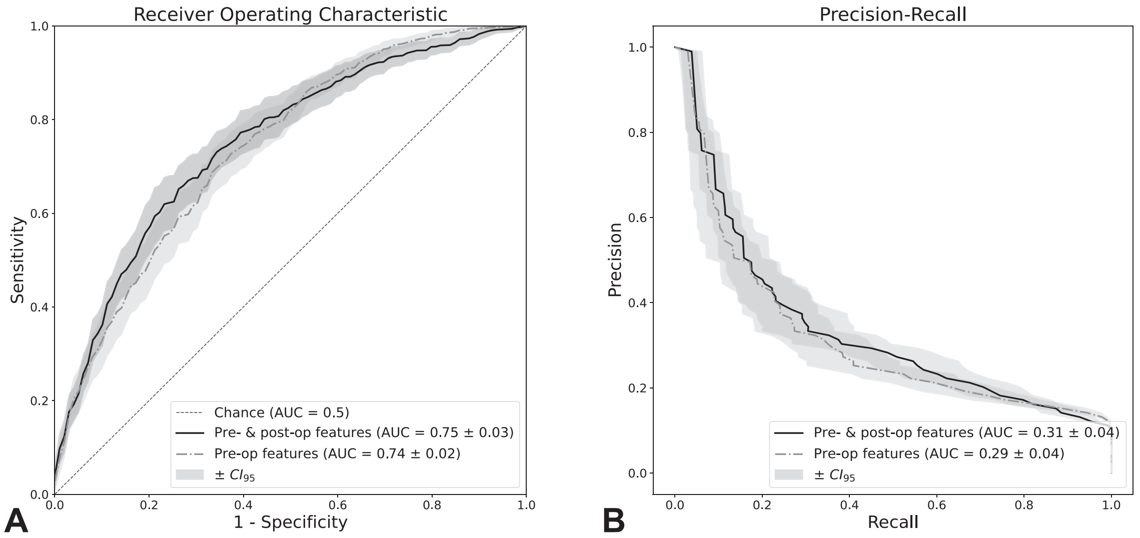

Figure S1 – Prognostic performance (ROC and PR-curve) for supratentorial cases

When considering only supratentorial cases (n=777), the models reach an AUC-ROC of 0.75 ± 0.03 (pre- & post-op) and 0.74 ± 0.02 (pre-op only). AUC-PR was scored at 0.31 ± 0.04 and 0.29 ± 0.04 respectively with a baseline value of 0.11.
