# Supplementary material for: Routine ICU Surveillance after Brain Tumor Surgery: Patient Selection Using Machine Learning
Source: J Clin Med. 2024 Sep 26;13(19):5747. doi: 10.3390/jcm13195747 (PMC11477277; doi:10.3390/jcm13195747)
Supplement: Supplementary file 1 [file jcm-13-05747-s001.zip › Figure S2.pdf]

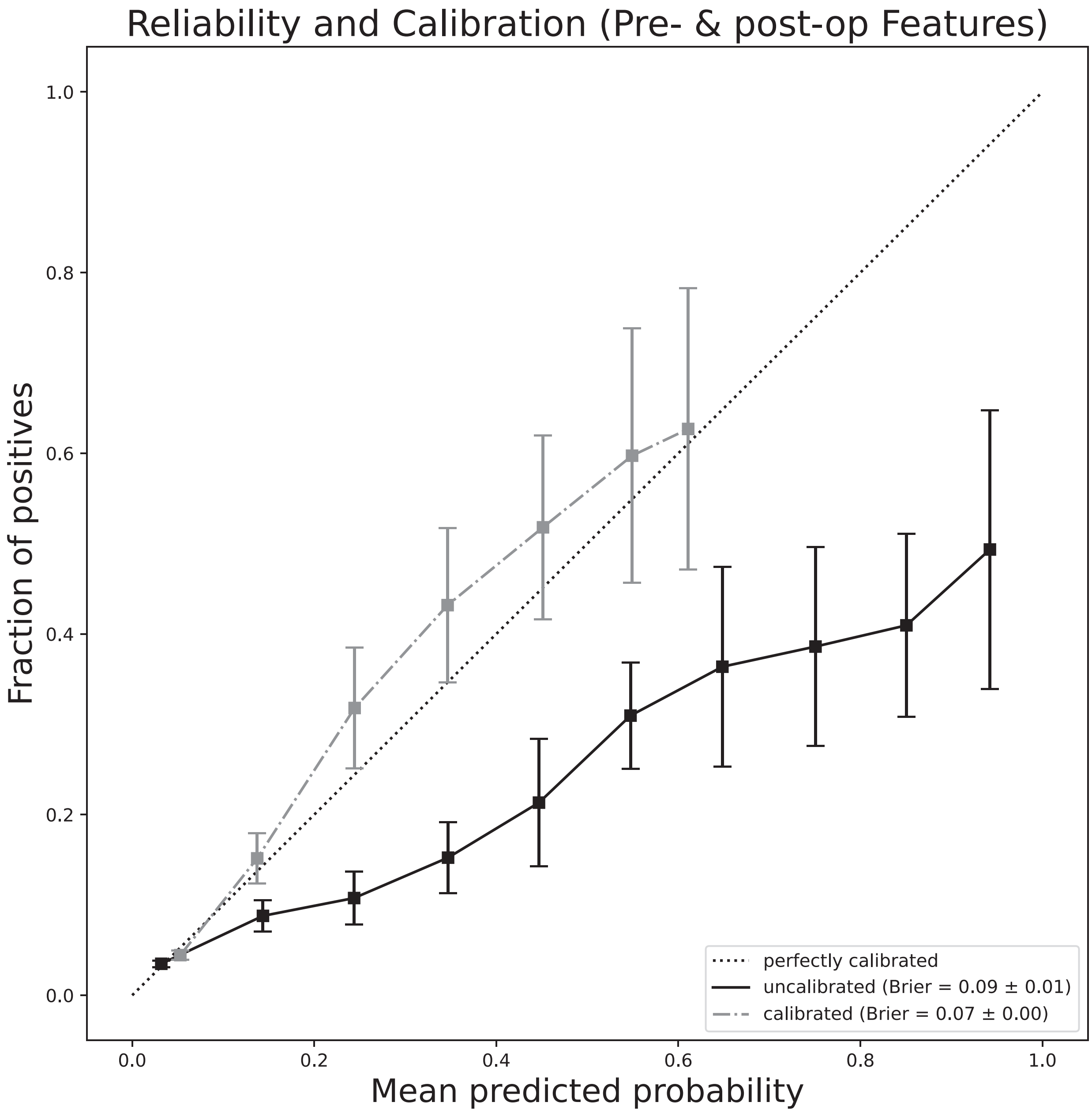

Figure S2 – Reliability and calibration

Boosted trees usually do not produce well-calibrated posterior probabilities. They are biased towards the center and avoid the extremes. The reliability plot of the raw model output for pre- & post-op features (black curve) shows a systematic overestimation in 5x5 cross-validation. Fitting an isotonic regressor that maps the output of the classifier to a calibrated probability in [0, 1] (grey, interrupted curve) shows a clear improvement of predicted probabilities after calibration in this manner.
