# Supplementary material for: Routine ICU Surveillance after Brain Tumor Surgery: Patient Selection Using Machine Learning
Source: J Clin Med. 2024 Sep 26;13(19):5747. doi: 10.3390/jcm13195747 (PMC11477277; doi:10.3390/jcm13195747)
Supplement: Supplementary file 1 [file jcm-13-05747-s001.zip › Figure S3.pdf]

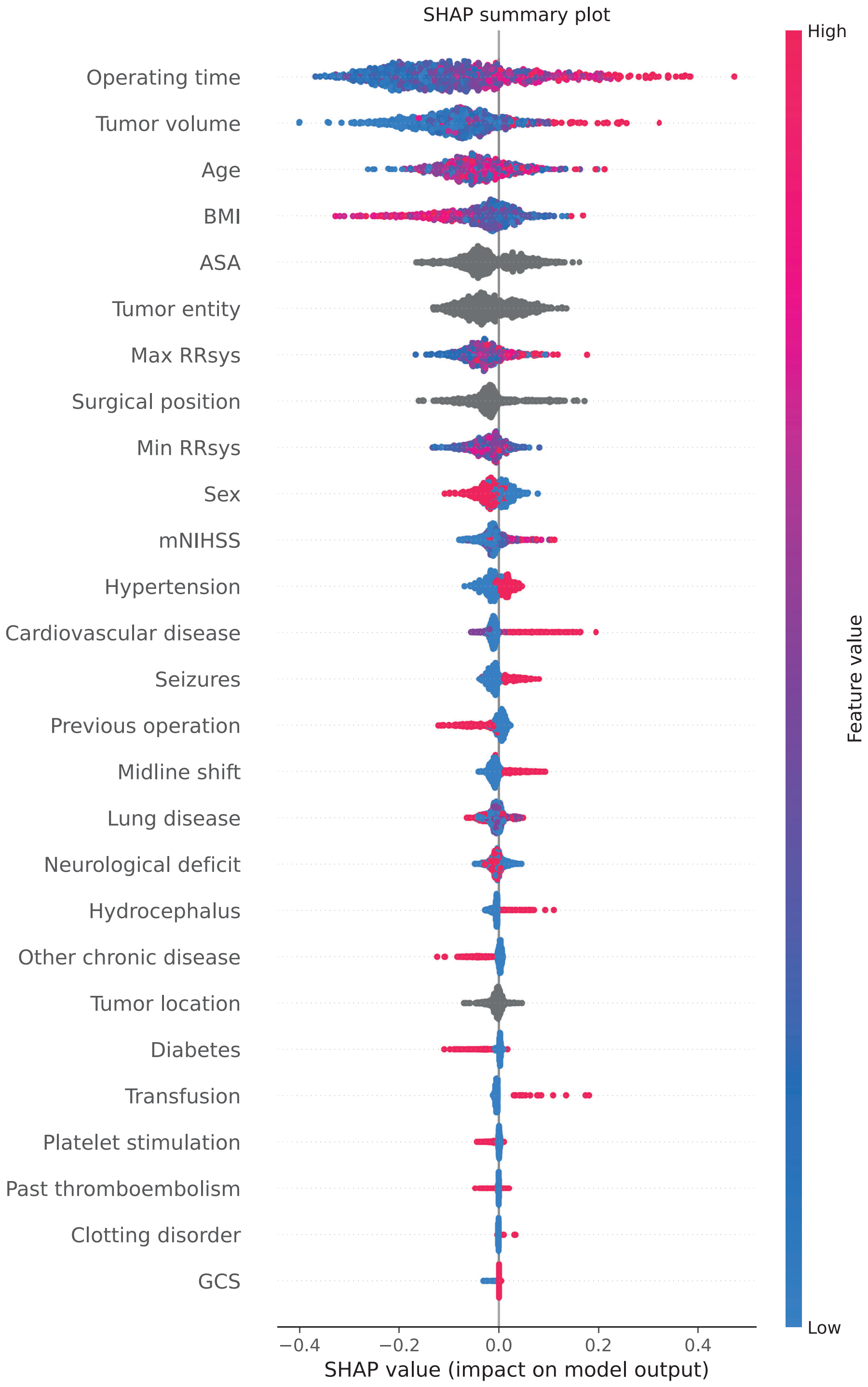

Figure S3 – Summary plot of SHAP values

Prognostic features are sorted by magnitude of SHAP value. Feature values for individual markers of each case are color-coded from low (blue) to high (red values). Dichotomic features were coded as one for "present" and zero for "non-present". Categorical features and the most important continuous variables are shown in more detail in fig. S4.
