# Supplementary material for: Routine ICU Surveillance after Brain Tumor Surgery: Patient Selection Using Machine Learning
Source: J Clin Med. 2024 Sep 26;13(19):5747. doi: 10.3390/jcm13195747 (PMC11477277; doi:10.3390/jcm13195747)
Supplement: Supplementary file 1 [file jcm-13-05747-s001.zip › Figure S4.pdf]

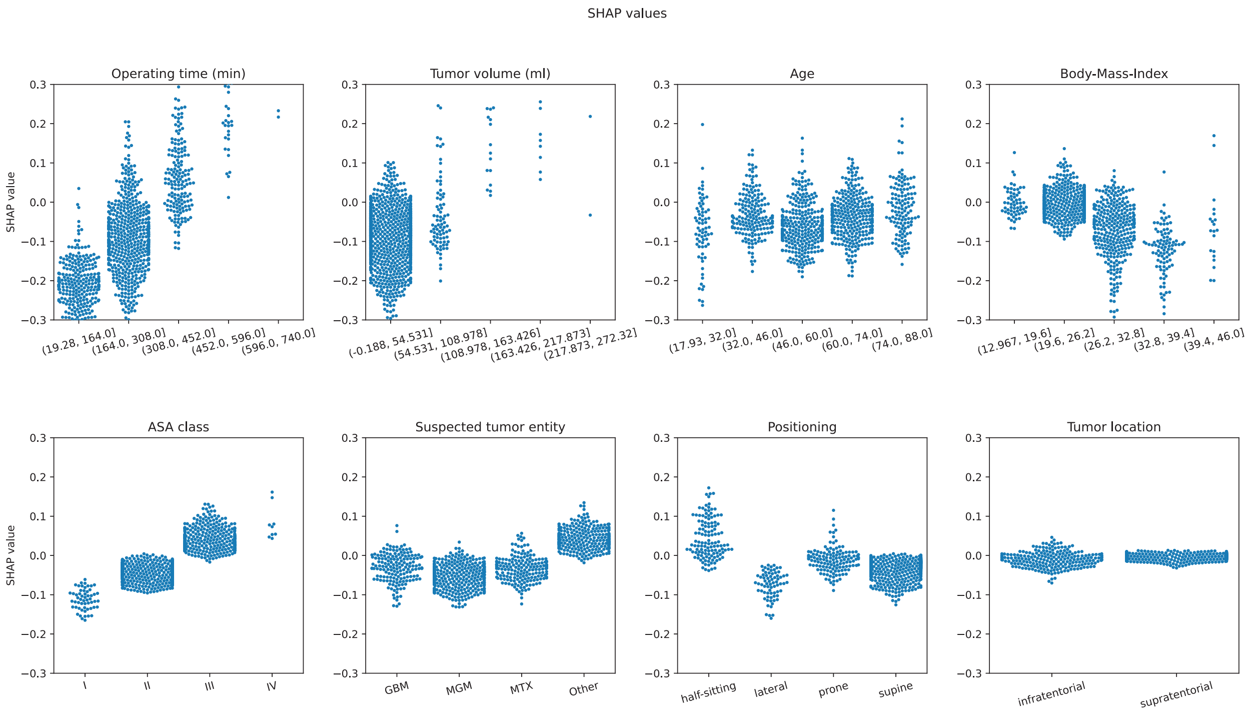

Figure S4 – SHAP values for selected features

While operating time and tumor volume show a strong linear relationship with SHAP values, patient age delineates a much weaker linear relationship. Interestingly, BMI-values above ideal but below manifest obesity yield negative SHAP values. ASA classes III and above will move the boosted trees towards an ICU decision as well more “rare” tumor entities and the half-sitting and prone positions.
